# Modified U-Net for liver cancer segmentation from computed tomography images with a new class balancing method

**DOI:** 10.1186/s42490-021-00050-y

**Published:** 2021-03-01

**Authors:** Yodit Abebe Ayalew, Kinde Anlay Fante, Mohammed Aliy Mohammed

**Affiliations:** 1 Department of Biomedical Engineering, Hawassa Institute of Technology, Hawassa University, Hawassa, Ethiopia; 2 Faculty of Electrical and Computer Engineering, Jimma Institute of Technology, Jimma University, Jimma, Ethiopia; 3 School of Biomedical Engineering, Jimma Institute of Technology, Jimma University, Jimma, Ethiopia

**Keywords:** Liver cancer, Segmentation, Deep learning, UNet

## Abstract

**Background:**

Liver cancer is the sixth most common cancer worldwide. It is mostly diagnosed with a computed tomography scan. Nowadays deep learning methods have been used for the segmentation of the liver and its tumor from the computed tomography (CT) scan images. This research mainly focused on segmenting liver and tumor from the abdominal CT scan images using a deep learning method and minimizing the effort and time used for a liver cancer diagnosis. The algorithm is based on the original UNet architecture. But, here in this paper, the numbers of filters on each convolutional block were reduced and new batch normalization and a dropout layer were added after each convolutional block of the contracting path.

**Results:**

Using this algorithm a dice score of 0.96, 0.74, and 0.63 were obtained for liver segmentation, segmentation of tumors from the liver, and the segmentation of tumor from abdominal CT scan images respectively. The segmentation results of liver and tumor from the liver showed an improvement of 0.01 and 0.11 respectively from other works.

**Conclusion:**

This work proposed a liver and a tumor segmentation method using a UNet architecture as a baseline. Modification regarding the number of filters and network layers were done on the original UNet model to reduce the network complexity and improve segmentation performance. A new class balancing method is also introduced to minimize the class imbalance problem. Through these, the algorithm attained better segmentation results and showed good improvement. However, it faced difficulty in segmenting small and irregular tumors.

## Background

Liver cancer is the sixth most common cancer worldwide. As of the Global Cancer Statistics report, it is the second and sixth cause of cancer death for men and women, respectively [1]. According to the WHO data, the percentage of liver cancer deaths in Ethiopia out of the total death in 2017 was about 0.16% [2]. In generall, there are two types of liver cancers, primary and secondary. Among primary types of cancers, hepatocellular carcinoma (HCC) accounts for 80% of the cases [3]. HCC is the third cause of cancer deaths and results in the death of around 700,000 people each year worldwide [4]. The major risk factors associated with primary liver cancers are cirrhosis resulting from alcohol usage, hepatitis B and C viruses, and a fatty liver disease caused by obesity [5]. Liver cancer can be diagnosed and detected by using different imaging tests like ultrasound, magnetic resonance imaging (MRI), and computed tomography (CT). From these, a CT scan is the frequently used imaging test [6].

A CT scan gives detailed cross-sectional images of the abdominal region. Most of the time further processing of these abdominal CT scan images is required to segment the liver and its counter tumorous areas from the rest of the CT image contents.

But still, the intensity similarity between the tumor and other nearby tissues in the CT images made the segmentation of the tumorous areas too difficult [5]. Therefore, these images need to be processed and enhanced to differentiate the cancerous tissue.

In a CT scan, the presence of liver cancer can be identified by the difference in pixel intensity in comparison to the surrounding healthy liver, i.e. the tumor area may be darker (hypodense) or brighter (hyperdense) than the surrounding healthy liver [7]. The manual segmentation of CT scan images is laborious and time-consuming for a clinical setting because of various factors, for instance, commonly the liver typically stretches over 150 slices in a CT volume, the shapes of the lesions are indefinite, the contrast between the lesions and the nearby tissue might be low, the shape and the size of the liver varies among patients and the intensity of the liver might be similar to the other organs [5, 8]. Considering these problems, researchers have designed different computer-aided diagnostic systems for the segmentation of liver and tumor from the abdominal CT scan images.

In earlier days, different traditional techniques were used to extract tumors from liver images. But these methods were not fully effective in the extraction of the tumor. Most of them are manual or semi-automatic and dependent on edge detectors rather than analyzing the image as a pixel. After hardware improvement in the 2000s, machine learning approaches came into a widely applicable system in image processing tasks like segmentation [9]. A variety of deep-learning methods have also been developed for automatic or semi-automatic segmentation of liver tumors. Among those, convolutional neural networks (CNN) are currently the most widely used method [10]. Researchers had used CNN and its extensions, fully connected layer and UNet, for liver and tumor segmentation.

Recent techniques for the segmentation of liver tumors can be classified into three classes according to the method that they implemented. These are convolutional neural networks (CNN), fully convolutional networks (FCN), and UNet convolutional networks. But CNN is the baseline for all methods.

The first method is the convolutional neural network (CNN). In this method, researchers had used pure CNN architectures for the segmentation of the liver and the tumor. In 2019, Budak et al. developed two cascaded encoder-decoder convolutional neural networks for efficient segmentation of liver and tumor. They proposed the EDCNN algorithm that includes two symmetric encoder and decoder parts. Each part consists of ten convolutional layers with batch normalization and ReLU activation followed by a max-pooling layer [11].

The other method is a fully convolutional network (FCN). FCN is an extension of CNN that substitutes the fully connected layer of CNN with a 1 × 1 convolution where the final output layer has a large receptive field that matches the width and height of the original image, enabling every pixel to be classified. FCNs have two parts, downsampling, and upsampling path. In the downsampling path, there are seven convolutional and five max-pooling layers that downsized the input image through convolution and max pooling operations. Researchers had also used this method for liver and tumor segmentation [12, 13].

The third method is the UNet convolutional neural network. UNet was designed for biomedical image segmentation by extending the work published in 2014 [14]. It works with small training samples and gives more accurate segmentation results. This network consists of a contracting path that extracts semantic or contextual information from the image and an expansive path which adds location information for each pixel and answers where each of them is localized. The two paths are more or less symmetric to each other, and yields a u-shaped architecture [15]. Researchers had been used this model for tumor segmentation by modifying and improving the architecture by increasing the depth of the structure and by adding more skip connections and dropout layers. And had combined it with other methods like graph cut and 3D conditional random fields for better segmentation results [8, 10, 16].

As of the available literature regarding U-Net, the maximum dice score obtained for liver and tumor segmentation is 0.9522 and 0.63 respectively. Additionally, Christ et al. and Chlebus et al. had used 3D post-processing methods for better segmentation results [8, 16]. But still, the segmentation performance was comparatively poor.

In this paper, a deep learning-based segmentation algorithm was employed for liver and tumor segmentation from abdominal CT scan images. The main contributions of this work are, first, it applied data augmentation tasks that solve the limitation of available data in biomedical images, second, it highly reduced the time needed for training by reducing the number of filters in each convolutional block thereby it reduced the number of trainable parameters and third it minimized the effect of class imbalance which presents between the tumor and the background through discarding slices with no tumor information from the datasets and used only slices with full information. These modifications improve the performance of the algorithm in detecting the tumor from the CT images. Finally, this work also showed the direct segmentation of liver tumors from the abdominal CT scan images without segmenting the liver first. By this, we were able to show the results of the three segmentation experiments in one paper, unlike others.

## Results

For training, three separate models with similar architectures had been used. The first model was trained using abdominal CT scan images with liver annotations for liver segmentation. Then the second model was trained using liver images with tumor annotations for the segmentation of the tumor from the liver. Finally, the third model was trained using abdominal CT scan images with tumor annotations for the segmentation of the tumor directly from the abdominal CT scan images.

**Fig. 1:**
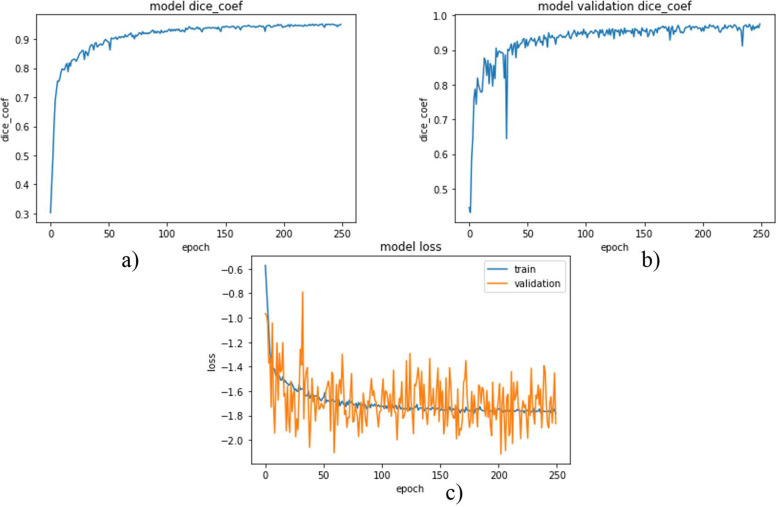
DSC and loss plot (**a**) and (**b**) are model DSC for training and validation data respectively and (**c**) is a model loss

**Fig. 2:**
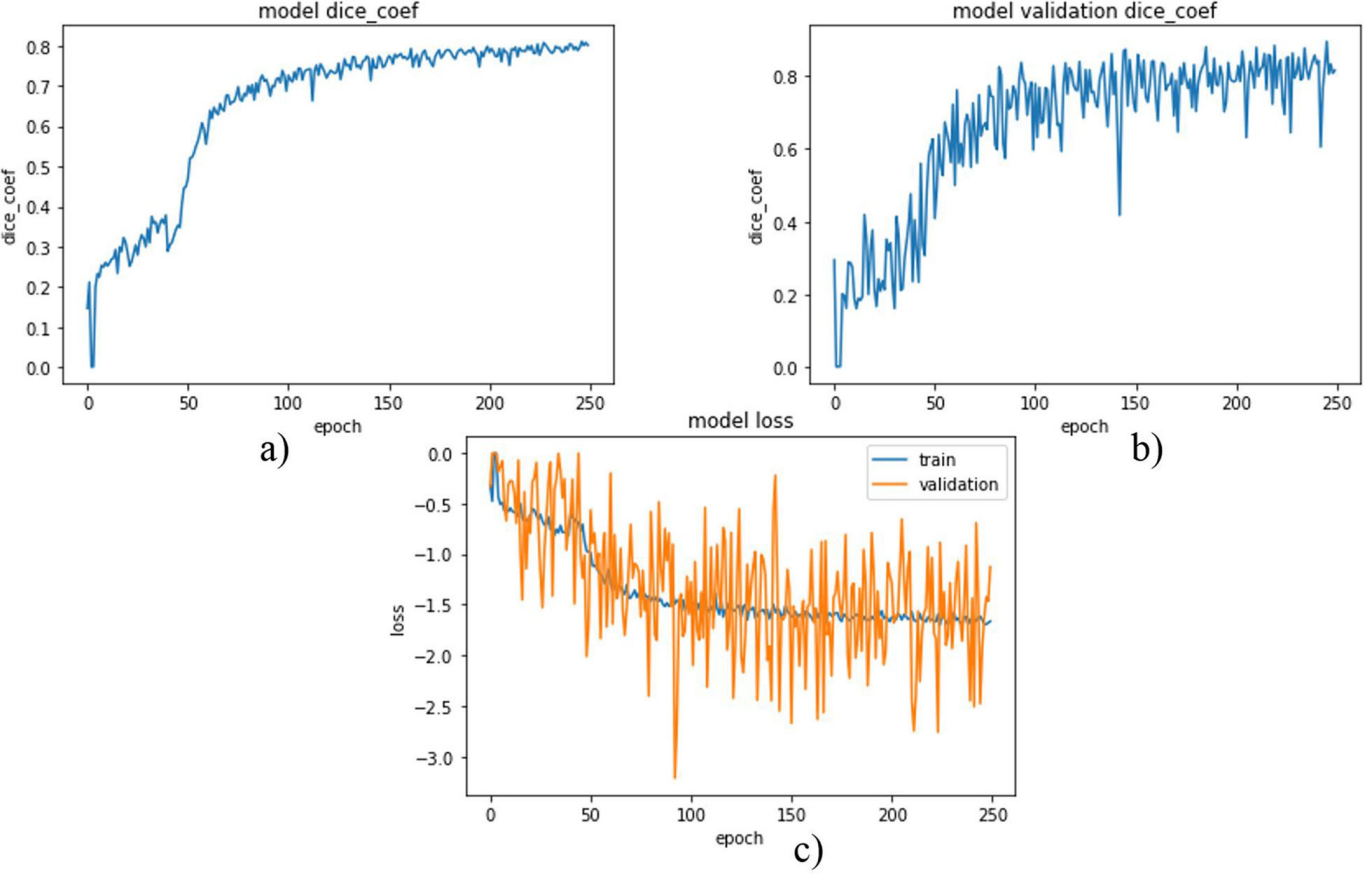
DSC and loss plot (**a**) and (**b**) are model DSC for training and validation data respectively where (**c**) is the model loss

**Fig. 3:**
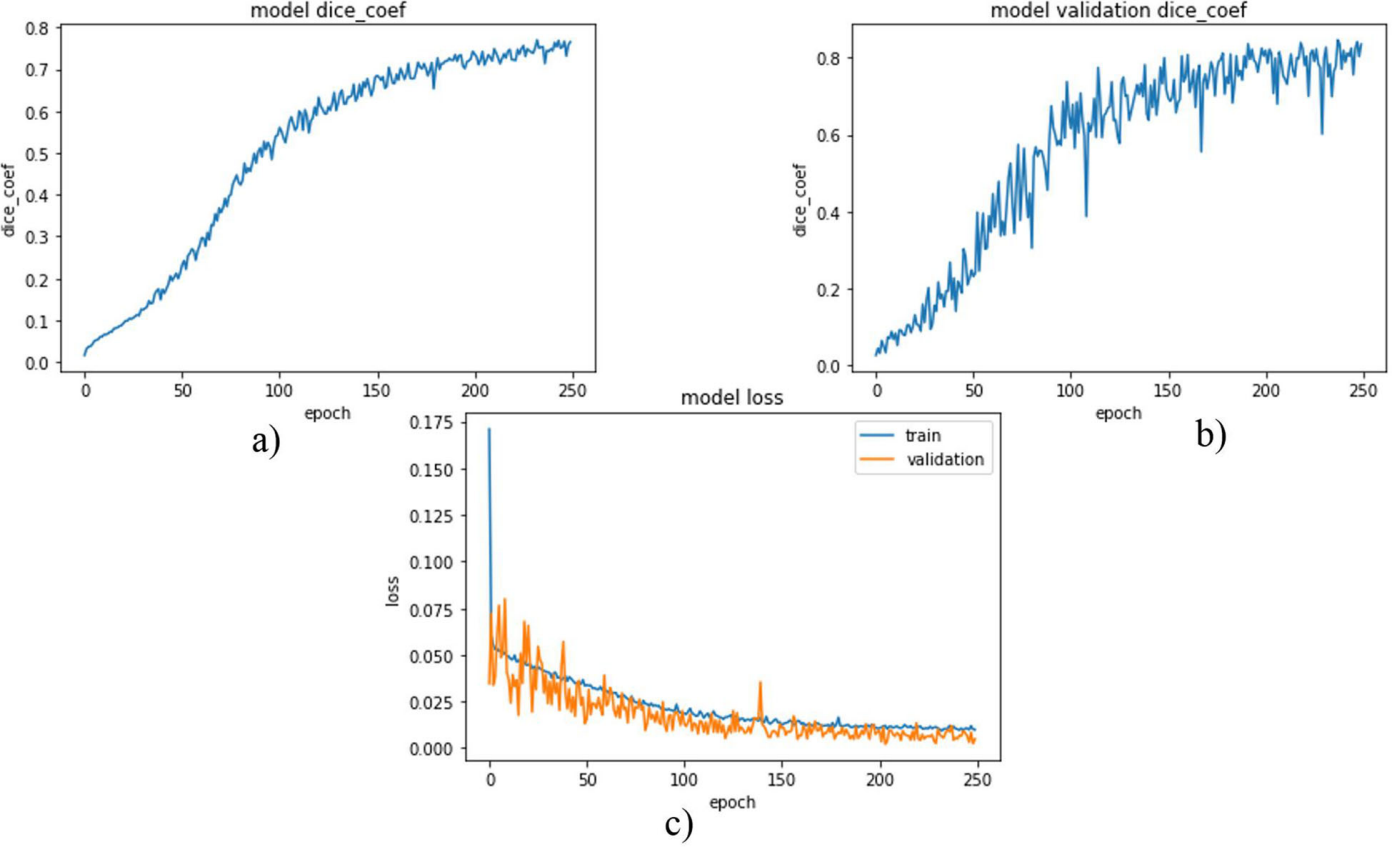
DSC and loss plot of the model (**a**) and (**b**) are model DSC for training and validation data respectively and (**c**) is a model loss

Each network was trained using 2346 images with data augmentation from scratch. Images were 512 × 512 in dimension. Since processing the whole images with these sizes is difficult due to limited GPU memory, the images were resized to a dimension of 128 × 128 even if degradation of image quality and information loss is inevitable. Weighted dice loss was chosen as a loss function for the first two networks and showed better performance during training. For the last model, which was trained to segment tumors directly from abdominal CT scan images, binary cross-entropy was chosen as a loss function and for all those three models Adam was selected as an optimizer through experiments. The network’s model DSC and model loss for liver segmentation, tumor segmentation from the liver, and tumor segmentation from the abdominal CT scan images were plotted from Figs. 1, 2 and 3.

The first two plots (a and b) in Fig. 1, show the model DSCs for training and validation data during the training of the model for liver segmentation. And the third plot in Fig. 1 shows the model losses for training and validation data.

And as it can be inferred from the two graphs, the model has good performance for both training and validation data. The DSC for both graphs increased highly around the first 100 epochs and its increment became gradual and been nearly constant. Finally, the DSCs became 0.9511 and 0.9633 for training and validation data respectively.

As observed from the third graph, the losses for both data decreased highly up to around the first 100 epochs and after that, it became nearly constant. The final losses for training and validation were − 1.7567 and − 2.1753 respectively.

As it can be inferred from the three graphs in Fig. 1, the model has good performance in segmenting the liver from the abdominal CT scan images.

As Fig. 2 (a) shows the model DSCs for the training data increased up to some point and became nearly constant. This showed the network was good during training. In the second plot (b), the model DSC for validation data was also plotted and some fluctuations were observed. At the last epoch, DSCs of 0.7769 and 0.8375 were obtained for training and validation data respectively.

In Fig. 2 (c) the model losses were plotted. The losses for the training and validation data decreased as expected and became nearly constant. And finally, losses of − 1.6291 and − 2.0278 were obtained for training and validation data respectively.

As shown in Fig. 3, model DSCs and model losses were plotted for tumor segmentation from the abdominal CT scan images. The first two plots are model DSCs for training and validation data. At the last epoch, DSCs of 0.7734 and 0.8240 were obtained for training and validation data respectively.

And in the third plot Fig. 3, the model losses for the two data were plotted. Here also some fluctuation in validation losses was observed. But the training loss decreased almost constantly. And obtained losses of 0.0093 and 0.001 for training and validation respectively.

### Test results for liver segmentation

**Fig. 4:**
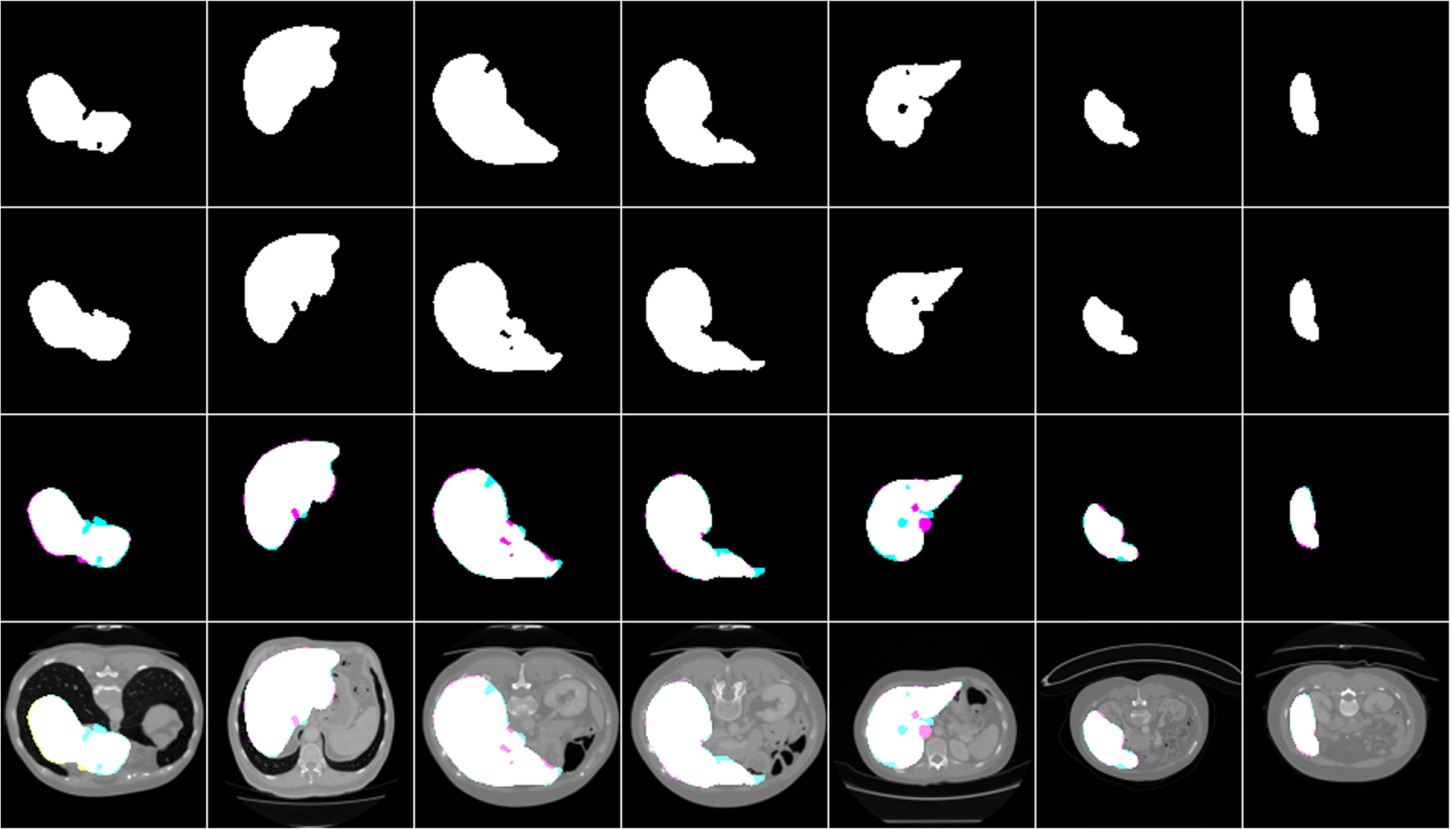
Liver segmentation results, ground truths, and overlap images

The performance of the liver segmentation algorithm was evaluated using different performance metrics and the result is included in Table 1. The segmentation result of the algorithm with the respective ground truth images is included in Fig. 4.

**Table 1:** Test results of others and our work for liver segmentation

Papers	Dice score	SVD	Accuracy
Christ et al. [12]	0.9430	_	_
Liu et al. [10]	0.9505	_	_
Budak et al. [15]	0.9522	_	_
**This work(U net)**	**0.9612**	**0.0388**	**0.9931**

Row 1 shows the result of the model, row 2 shows the respective masks, row 3 shows overlap images of the result with a mask, and row 4 shows both results and mask on the original CT scan image. As shown in Fig. 4 the liver segmentation result is satisfactory and the algorithm could almost segment the liver from the abdominal CT scan images fully. It has an average dice score of 0.96 which is greater than the others by 0.01. But in some cases, it missed some portion of the liver as it is shown with cyan and segmented nearby tissues as a liver as it is shown as magenta in row 3.

### Test results for tumor segmentation

**Fig. 5:**
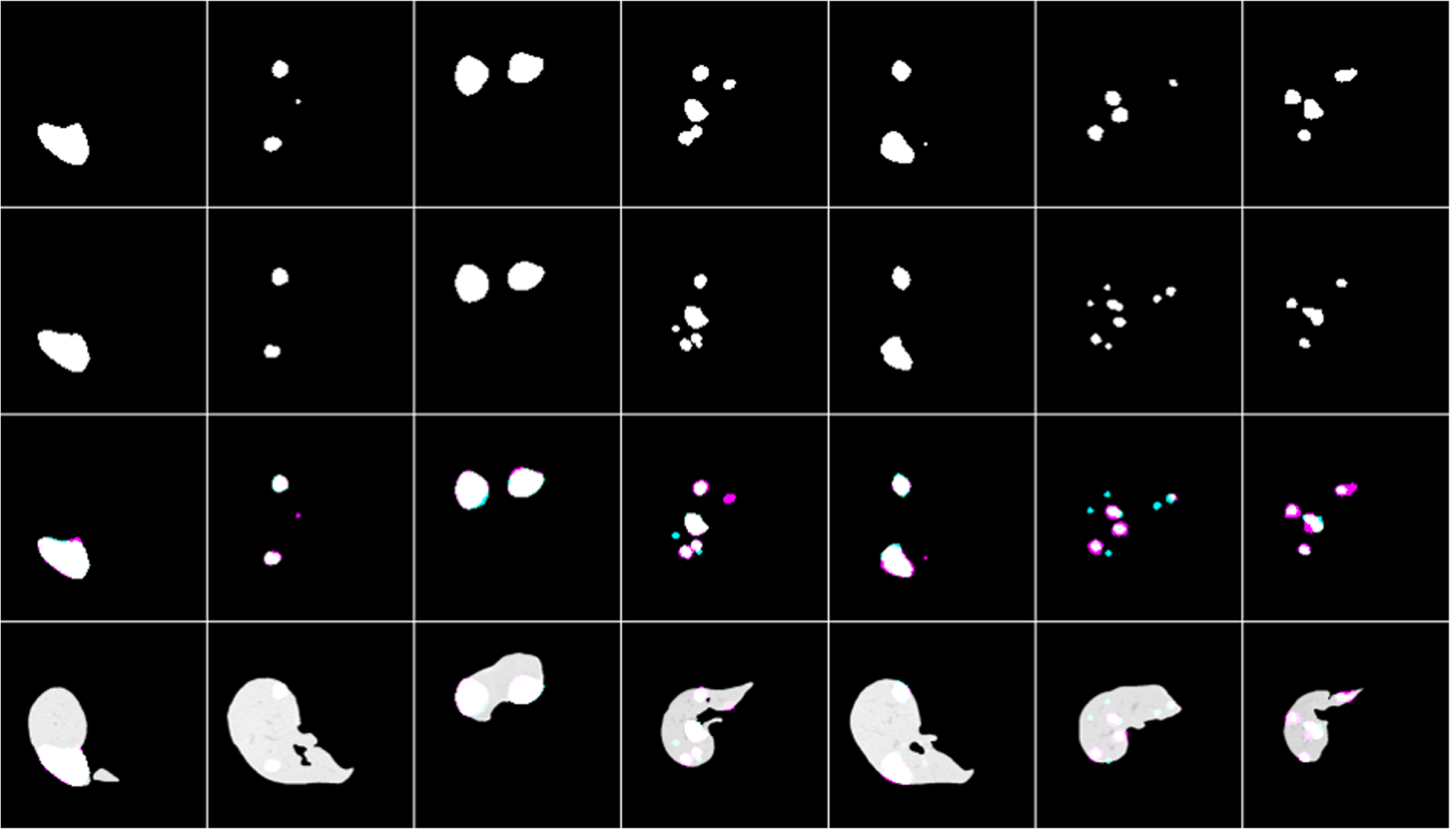
Tumor segmentation results from the liver with the respective masks and overlap images

**Fig. 6:**
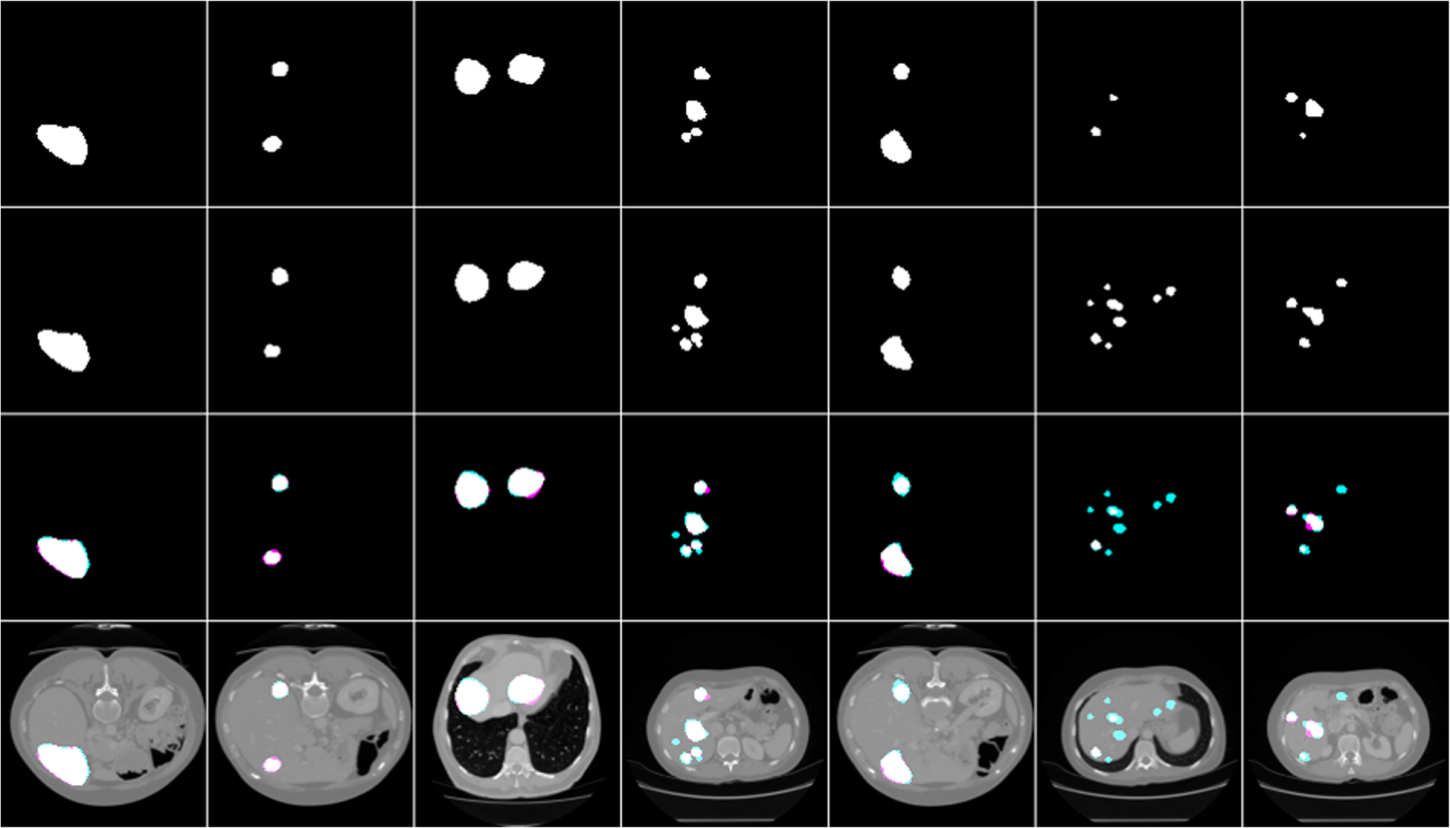
Result of tumor segmentation from abdominal CT images with the respective masks and overlap images

The segmentation result of this network on segmenting liver tumors from the liver and directly from the abdominal CT scan images was evaluated using different performance metrics and the result is included in Table 2. The segmentation result of the algorithm with the respective ground truth images is included in Figs. 5 and 6.

**Table 2:** Test results of others and our work for tumor segmentation

Papers	Dice score	SVD	Accuracy
Chlebus et al. [13]	0.58	_	_
Budak et al. [15]	0.63		
**This work (U net) from liver**	**0.74** ± **0.02**	**0.26** ± **0.02**	**0.9954**
**This work (U net) from abdominal CT image**	**0.63** ± **0.02**	**0.37** ± **0.02**	**0.9950**

Row 1 shows the result of the model, row 2 shows the respective masks, row 3 shows overlap images of the result with a mask and row 4 shows both results and mask on the liver.

As shown in Fig. 5, the algorithm has good segmentation ability on circular tumors and could also detect the distributed tumors from the same liver slice. It has an average dice score of 0.74, which is greater than the others by 0.11. But in some cases, it failed to segment some tumors as it is seen as cyan and segmented other tissues as tumor as it is seen as magenta in row 3.

Row 1 shows the result of the model, row 2 shows the respective masks, row 3 shows overlap images of the result with a mask and row 4 shows both results and mask on the abdominal CT image.

As shown in Fig. 6, the tumor segmentation directly from the abdominal CT scan image showed good performance relative to works done by other researchers. It has a relatively similar performance with those works that segment the tumor with a two-way process. It has an average dice score of 0.63. But it failed to segment some tumors as it is seen as cyan in row 3 and it also segmented other nearby tissues as a tumor as it is shown with magenta in row 3.

## Discussion

In the original UNet paper the batch size of 1 was used for maximum usage of GPU memory without considering the time it took for training [15]. As the batch size decreases the training time will increase and the probability of using maximum GPU memory increases. Therefore the selection of batch size needs great care. Unlike [15], in this work batch size of 8 was used that compensates both GPU memory problems and training time after many trials. That means the network was trained using eight images at a time.

To test the liver segmentation performance of the developed network, 392 images were used. And those images were preprocessed using the same preprocessing technique that was implemented on the training data. The result of the network was evaluated using the respective ground truths of the images and the comparison result of this algorithm with works of Christ et al. who had used a cascaded deep neural network with 3D conditional random fields to segment the liver and its lesions [16], Liu et al. who came up with GIU-Net that combines the improved UNet with the graph cut algorithm for segmenting liver sequence images [10] and lastly with Budak et al. who developed two cascaded encoder-decoder convolutional neural networks for the segmentation of liver and its tumor [11], were also included.

Table 1 shows the result obtained from this work and other works.

For testing the segmentation ability of the developed algorithm on segmenting the liver tumor a total of 392 images with their respective ground truths were used. The tumor was segmented in two ways. The first is the segmentation of the tumor directly from the abdominal CT scan image and the other is from the liver after segmenting it first. The result of the network was evaluated using the respective ground truths of the images and the comparison result of this algorithm with works of Chlebus et al. who used UNet by modifying it with object-based post-processing to segment liver tumor [8], and Budak et al. who implemented an encoder-decoder convolutional neural network for liver tumor segmentation [11], were also included.

Table 2 shows the tumor segmentation result of two papers and the current work.

This algorithm highly reduces the complexity of the network by reducing the number of filters in each convolutional block. This decreases the time needed to train the network from a few hours to 40 min. In this thesis, 2346 images with data augmentation were used to train the network which is very small when it is compared with other works that had used more than 20,000 images.

And here the class frequency difference between the liver and the background was minimized through removing the CT slices with no liver that affects the segmentation performance in addition to introducing a weight vector to the loss function. The result of this algorithm was compared with other works to show how this algorithm improves liver segmentation performance.

And the work also shows a new way for liver tumor segmentation. It can segment the tumor directly from the abdominal CT scan images, unlike the others which followed two steps to segment it. In other work, to segment liver tumors, the liver has to be segmented first and then the tumor segmentation precedes next to that.

But this work came up with segmenting of the tumor directly without liver segmentation and by this, a comparable segmentation result of 0.63 DSC was obtained. In addition to this, the tumor segmentation was also done using the previous way. That means by following a two-step process like others and obtained a DSC of 0.74 that differs by an average of 0.11 from the previous works. Chlebus and his colleagues had used the post-processing method, which includes 3D connected components and random forest classifiers. However, the segmentation result obtained from this algorithm is greater than them by 0.16. This improvement is due to the class balancing that the work implemented. As discussed above, the class balancing was done by removing slices with no tumor. The difference between the numbers of tumor pixels to background pixels largely affects the segmentation result. Therefore this work tried to decrease this class imbalance by removing those slices with no tumor from the whole dataset in addition to the weight factor added to the loss function and observed a performance improvement. The segmentation result is compared with other works to show the improvements in liver tumor segmentation.

### General results of the architecture

This segmentation algorithm highly improves the efficiency of liver tumor segmentation. First, it reduces the complexity of the network by reducing the number of filters needed on each convolutional block that decreases the number of trainable parameters. Due to this the time needed for training the network greatly reduced. The total time needed to train the network for 250 epochs is about 40 min on Kaggle kernel. This is a great achievement in deep learning-based segmentation in which the time and complexity of the network matter a lot.

And the other burning issue in deep learning-based segmentation was the absence of enough training samples to train the network. And this is also solved by the developed algorithm. It only needs small training samples and used excessive data augmentation. By this, it can increase the number of training samples present. Data augmentation applied affine deformations on those available images that helped the network to learn invariance to those deformations hence, deformation is the most common variation in biomedical images.

**Fig. 7:**
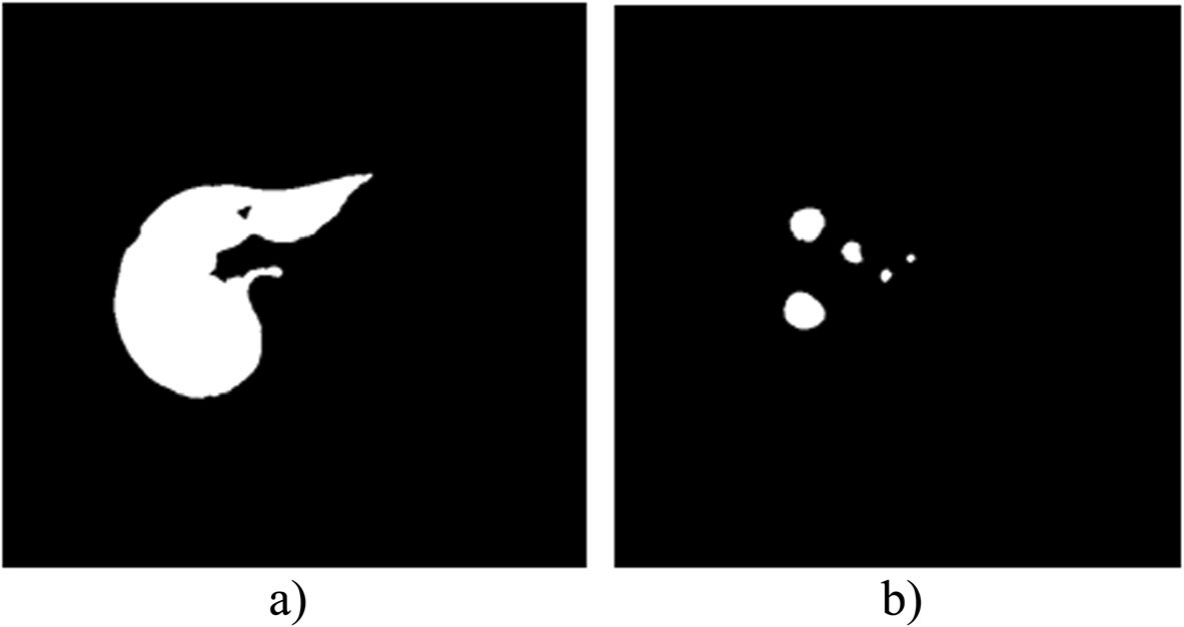
The class imbalance between liver and tumor with the background. (a) shows the liver and (b) shows the tumor

And the other important thing that should be considered during liver tumor segmentation or other biomedical image segmentation is the class imbalance between the two classes to be segmented. There is a large difference in size between the tissue to be segmented and the background. This highly affects the segmentation performance. For example, in Fig. 7 the number of white pixels to black pixels shows a high difference.

The ratio of white pixels to black pixels can be calculated using Eq. 1. It is 1: 9 and 1: 85 for liver and tumor masks respectively. Due to this, the network gets more black pixels than white pixels and learns from that during training. Its probability of learning from white pixels is very small when it is compared with the black ones. This results in poor performance of the network.

1 $$ \mathrm{Ratio}=\frac{Number\ of\ white\ pixels}{\  number\ of\ black\ pixels} $$

On the original UNet paper, the authors included a weight map that pre-computed from the ground truth images for balancing the class frequencies. In addition to this, in this paper, it is reduced using the removal of slices with no tumor information. During data preparation, the first step was checking all patients’ data with tumor from both datasets. Then remove data that is obtained from healthy ones from the dataset and next search and remove for slices with no tumor. Lastly, the data with the tumor only was arranged and saved sequentially.

The network had been trained using those data and it’s observed that the network’s performance showed a great difference. The network performance increased with 0.01 and 0.11 for liver and tumor respectively.

This work also introduces a new way for tumor segmentation. Before this work, tumor segmentation has been done from the liver after segmenting it first from the abdominal CT scan image. The segmentation was a two-way process. But here liver tumors can be detected and segmented directly from the abdominal CT scan images with relatively comparable performance. This decreases the time and the effort needed during the segmentation of the tumor.

Experiments were done to show the effect of filter reduction and the application of data augmentation on the overall model performance.

Table 3 demonstrates the results of the models with an original and reduced number of filters and their performance before and after applying a data augmentation.

**Table 3:** Experimental results for liver and tumor segmentation with filter size reduction and application of data augmentation

	Liver (In DSC)	Tumor from liver (In DSC)	Tumor from abdominal CT image (In DSC)
UNet (with original filter size)	0.9529	0.7384	0.6743
Modified UNet without data augmentation	0.9027	0.0992	0.0287
Modified UNet with data augmentation.	0.9612	0.74	0.63

As Table 3 shows, reducing the filter size didn’t reduce the model’s performance, rather it shows small improvements in both liver and tumor segmentation, and the training time is also reduced by about 1/3. The model performance is checked with and without data augmentation. Without data augmentation, it shows overfitting. It was good during the training but it is worse at the testing time especially for tumor segmentation since most of the tumors are very small.

Even if the algorithm showed good improvement on liver and tumor segmentation, it still fails to segment correctly in some slices. In liver segmentation, the algorithm almost segments the liver correctly but it fails in some slices in which the full liver is not captured and in slices in which the liver is covered by other overlapping organs and seems to be divided into parts. Regarding tumor segmentation, the algorithm mostly fails to segment tumors that are small and irregular in shape.

## Conclusion

This paper focused on segmenting the liver and its tumor using a deep learning method. The method consists of three modified UNet models for the liver, the tumor from the liver, and the tumor from abdominal CT scan image segmentation. Using this algorithm a DSC of 0.96 and 0.74 for the segmentation of liver and tumor from the liver respectively were attained which showed an improvement of around 0.01 and 0.11 respectively. This improvement was obtained due to the reduction of the class imbalance that occurred in the data manually by removing unnecessary images and the selection of good hyperparameters through many trials.

## Method

### Description of materials

#### Datasets

Images that were used to train and test liver and liver cancer segmentation algorithm developed by this paper were taken from two publicly available datasets, 3Dircadb01 (3D Image Reconstruction for Comparison of Algorithm Database) [17] and LITS (Liver Tumor Segmentation) Challenge [18]. The 3DIRCADb dataset is challenging to utilize since there is a high variety of data and the liver and tumor complexity [11]. Detailed information about the two segmentation datasets is included in Table 4.

**Table 4:** Liver and tumor segmentation data sets

Dataset	Number of patients	Image size	Pixel width and height	Slice thickness	Pixel spacing	Slice number	Tumor data out of 100%
3D-IRCADb01	20	512 × 512	0.56–0.87 mm	1–5 mm	0.55–0.95 mm	74–260	75%
LITS	131	512 × 512	–	0.7-5 mm	–	–	63%

Table 4 shows detailed information about the two datasets.

#### Data preparation

Images taken from the two datasets should be prepared to use them for training and testing the developed algorithm. The 3D-ircadb01 dataset contains up to seven folders under each patient’s data for the tumor masks depending on the anatomical position of the tumor on the liver. Therefore these tumor masks from those different folders should be added and put into one folder since the main intention is on the segmentation result not on the tumor’s anatomical position.

And the images in the LITS datasets are three-dimensional and there is no separate mask for the liver and its tumor. Instead, they are found on the same mask image under the segmentation folder in the dataset. Since the developed algorithm is two dimensional (2D), the data should be converted into 2D. A separate mask for the liver and tumor must also be prepared. This data preparation was done using an ImageJ tool. From both datasets, the patient data with no liver and tumor masks are discarded. And from each patient data, images or slices which are taken at the starting and ending of scanning, with no liver information were also discarded for reducing the class imbalance present between the background and foreground.

The number of images used for training and testing is included in Table 5.

**Table 5:** Training and testing images

Training images	Testing images
2346 + Data augmentation	392

#### Image preprocessing

The images were 512 × 512 in dimension originally. Using those images as it was is difficult due to limited GPU memory. Therefore, all images were resized with a factor of 0.25. And the images were also normalized to have a value between 0 and 1.

#### Computing platforms

The acquired images from both publicly available datasets were processed and analyzed on Kaggle. Kaggle is an online community of data scientists, owned by Google that provides cloud infrastructures such as a built-in Python Jupyter notebook, graphical processing unit (GPU), tensor processing unit (TPU), and data storage platform for facilitating the works of data scientists.

### The segmentation algorithm

The algorithm is based on the UNet architecture developed by Ronneberger et al. in 2015. This algorithm includes two 2D UNet architectures, for the liver and its tumor. These architectures were designed to segment liver and tumors from the abdominal CT scan images.

#### Network architecture

For both liver and tumor segmentation, the same U shaped network architecture is used. It consists of a contracting path, an expansive path, and a bottleneck part like the original UNet. But, here in this paper, after each convolutional layer, batch normalization is added in all three parts of the network and a 0.5 dropout layer is added after each convolutional block of the contracting path.

**Fig. 8:**
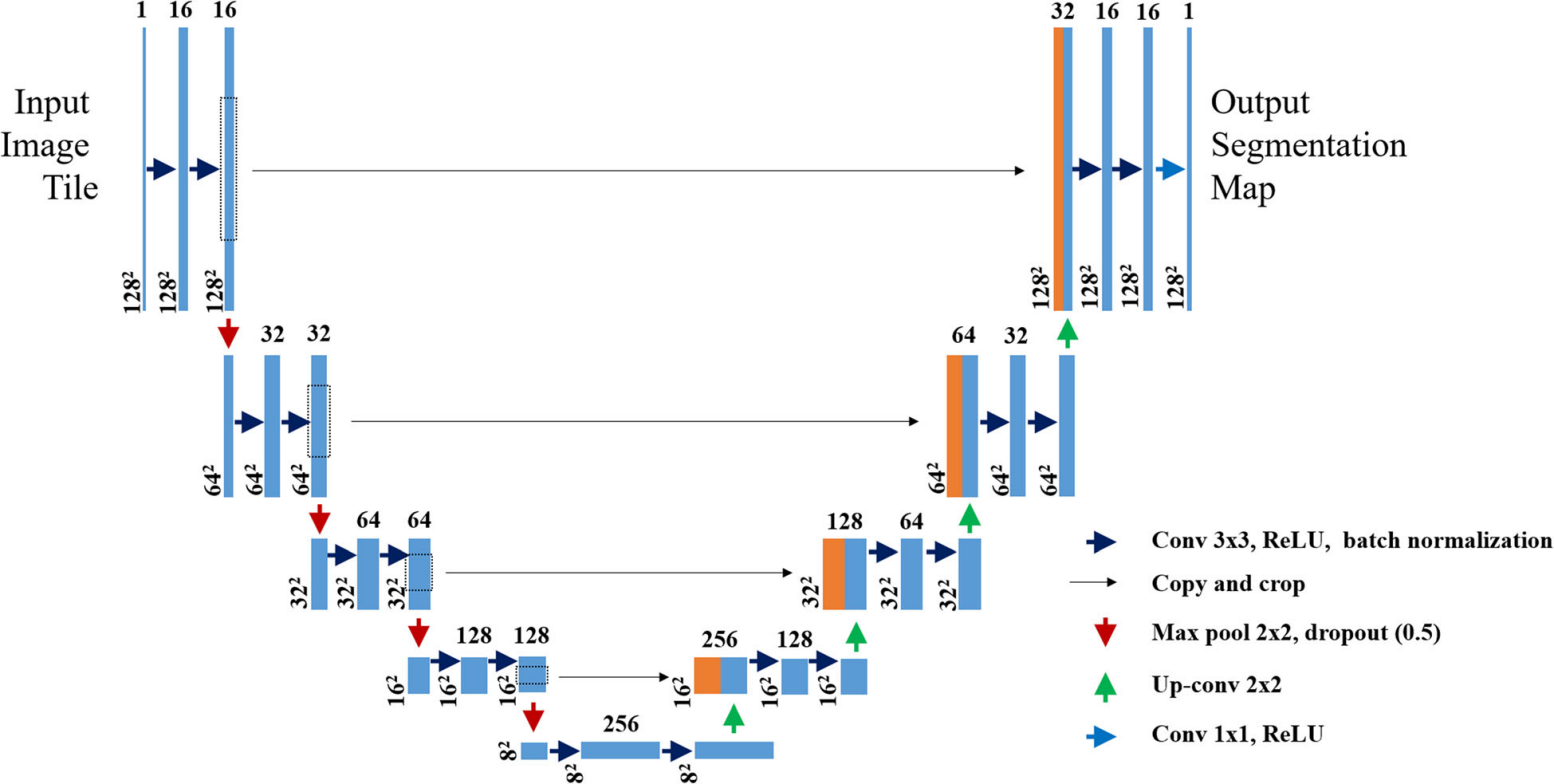
The network architecture

The batch normalization is important for normalizing the outputs of the convolutional layers to have a mean of zero and a standard deviation of one and the dropout layer randomly deactivated some neurons in the hidden layer to prevent overfitting of the network. And the other modification is done on the number of filters of each convolutional block. In the first block, there are 16 filters and it will be doubled in the consecutive three blocks and become 128 at the last convolutional block. The details of the modified network architecture are indicated in Fig. 8.

##### Contracting or downsampling path

The contracting path also called encoder is composed of 4 blocks. Each block is composed of:

- 3 × 3 Convolution Layer with ReLU activation function and batch normalization
- 3 × 3 Convolution Layer with ReLU activation function and batch normalization
- 2 × 2 Max Pooling
- Drop out layer (0.5)

The purpose of this contracting path is to capture the context or semantics of the input image to be able to do segmentation. It extracts features that contain information about what is in an image using convolutional and pooling layers. During this process, the size of the feature map gets reduced and the deep or high-level features of the image will be obtained but the network loses the spatial or location information in which those features are found.

##### Bottleneck

This part of the network is between the contracting and expanding paths. The bottleneck is built from two convolutional layers with batch normalization.

##### Expanding or upsampling path

The expanding path also called decoder is composed of 4 blocks. Each of these blocks is composed of

- Up convolution or Deconvolution layer with stride 2.
- Concatenation with the corresponding cropped feature map from the contracting path.
- 2 (3 × 3 Convolution layer with ReLU activation function and batch normalization).

The purpose of this expanding path is to recover the feature map size and to add spatial information for the segmentation image, for which it uses up-convolution layers.

The course contextual information from the contracting path will be transferred to the upsampling path through skip connections.

##### Skip connections

There could be a loss of low-level information during the decoding process. To recover this information lost and to let the decoder access the low-level features produced by the encoder layers skip connections are used. Intermediate outputs of the encoder are concatenated with the inputs to the intermediate layers of the decoder at appropriate positions. This enables precise localization combined with contextual information from the contracting path.

Details of the network architecture and layers found in each part of the model are shown in Fig. 8.

#### Training

The network architecture is based on the original UNet architecture. However, additional batch normalization and dropout layers are included in the network architecture of this work, and the number of filters in each convolutional block is also reduced. Therefore, training the network from scratch is needed. The input images and their corresponding segmentation masks are used to train the network. 2346 images from the two datasets with data augmentation were used. During training, many experiments were done by tuning the hyperparameters used in the network. Learning rate, batch size, number of epochs, and number of filters, validation split, dropout value, optimizer, loss function, and activation function had been checked for different values and assignments. After many trial and error a batch size of 8, epochs of 250, validation split of 0.30, and a dropout of 0.5 had been used.

##### Optimizer

Adam Optimizer is an extension for the stochastic gradient descent (SGD) and RMSprops (root mean squared). It is a method for efficient stochastic optimization that only requires first-order gradients with little memory requirements. It finds individual adaptive learning rates for each parameter in the network. Its name is derived from adaptive moment estimation [19]. In this work, Adam optimizer with a learning rate of 0.0001 had been used.

##### Loss function

Weighted dice loss and binary cross-entropy were used as loss functions to measure the variations of the predicted values from the actual values during the training of the network. The equations used for calculating weighted dice loss and binary cross-entropy are given in Eqs. 2 and 3 respectively.

2 $$ \mathrm{Loss}=-\mathrm{W}\left(\frac{2 TP}{2 TP+ FP+ FN}\right) $$

Where TP is true positive, FN is a false negative, FP is false positive and W is a weight factor that is introduced to balance the class frequency difference between the foreground and the background.

3 $$ \mathrm{BCE}=\frac{-1}{N}{\sum}_{i=1}^N\kern0.5em {y}_{i\cdot}\mathit{\log}\kern0.5em \mathit{\log}\left(p\left({y}_i\right)\right)+{\left(1-{y}_i\right)}_{\cdot}\mathit{\log}\kern0.5em \mathit{\log}\left(1-p\left({y}_i\right)\right) $$

Where BCE is binary cross-entropy, N is the total number of pixels, *y*_*i*_ is the predicted label for each pixel i, and *p*(*y*_*i*_) is the predicted probability of each pixel being foreground or background.

##### Data augmentation

Data augmentation is important to train the network effectively when there are small training samples available. In biomedical image segmentation tasks, there are often very little training data available. Therefore excessive data augmentation by applying affine deformations to the available training images is used. This allows the network to learn invariance to such deformations.

Data augmentation is specifically essential for biomedical image segmentation in which deformation is the basic difference in tissues. Less number of training pairs results in overfitting [15].

In the proposed work, in place or on the fly data augmentation technique had been used [20]. This type of augmentation artificially increases the size of the dataset by applying real-time data augmentation. In each epoch new randomly augmented data were given to the model. This increases the amount of data and the generalizability of the model.

### Performance metrics

For evaluating the performance of the segmentation method, the binary mask of the segmentation result is compared to the ground truth mask and their similarity is estimated. Different performance metrics like DSC, Jaccard similarity coefficient (JSC), accuracy, and symmetric volume difference (SVD) are used.

#### Dice similarity coefficient (DSC)

It measures the overlap between two binary masks. It is the size of the overlap of the two segmentations divided by the total size of the two objects. It ranges from 0 (no overlap) to 1 (perfect overlap). It represents the overall performance of the segmentation [21, 22]. It is calculated using Eq. 4.

4 $$ \mathrm{DSC}=\left(\frac{2 TP}{2 TP+ FP+ FN}\right) $$

Where TP is a true positive, FN is a false negative, and FP is a false positive.

#### Jaccard similarity coefficient (JSC)

It measures the similarity between the segmented image and the binary mask. It is the ratio of the intersection of two binary masks to their union [22]. It is given by Eq. 5.

5 $$ \mathrm{JSC}=\left(\frac{TP}{TP+ FP+ FN}\right) $$

Where TP is a true positive, FN is a false negative, and FP is a false positive.

#### Accuracy

Accuracy represents the ratio of correctly segmented samples to the total samples. It is approximately one for good segmentation results. It is calculated using Eq. 6 [12].

6 $$ \mathrm{Accuracy}=\frac{TP+ TN}{TP+ TN+ FP+ FN} $$

Where TP is a true positive, TN is a true negative, FN is a false negative, and FP is a false positive.

#### Symmetric volume difference

SVD is a measure of difference that exists between the segmented images with the ground truth. For good segmentation results, SVD approximates to zero. It is given by Eq. 7.

7 $$ \mathrm{SVD}=\left(1-\mathrm{DSC}\right) $$

Where DSC is the Dice similarity coefficient.

- True Positive (TP): denotes all pixels belongs to the foreground and classified as foreground.
- True Negative (TN): denotes all pixels belongs to the background and are classified as background.
- False Negative (FN): denotes foreground pixels that are incorrectly classified as background pixels by the classifier.
- False Positive (FP): denotes background pixels that are incorrectly classified as foreground pixels by the classifier.

## Data Availability

Not applicable.

## Abbreviations

2D: Two dimensional
3D: Three dimensional
BN: Batch normalization
CT: Computed tomography
DSC: Dice similarity coefficient
FCN: Fully convolutional network
GPU: Graphical processing unit
HCC: Hepatocellular carcinoma
IRCAD: Image Reconstruction for Comparison of Algorithm Database
JSC: Jaccard similarity coefficient
LITS: Liver Tumor Segmentation
MRI: Magnetic resonance imaging
ReLU: Rectified linear unit
SVD: Symmetric volume difference
TPU: Tensor processing unit
